# Tubular functional capacity and maladaptive parathyroid hormone response in early-stage chronic kidney disease

**DOI:** 10.17305/bb.2025.13395

**Published:** 2025-12-26

**Authors:** Branislava Ilinčić, Radmila Žeravica, Romana Mijović, Esma R Isenović, Dragan Burić, Dragana Žuvić, Velibor Čabarkapa

**Affiliations:** 1 Department of Pathophysiology and Laboratory Medicine, Faculty of Medicine, University of Novi Sad, Novi Sad, Serbia; 2 Center for Laboratory Diagnostic and Nuclear Medicine, University Clinical Centre of Vojvodina, Novi Sad, Serbia; 3 Department of Radiobiology and Molecular Genetics, VINČA Institute of Nuclear Sciences-National Institute of the Republic of Serbia, University of Belgrade, Belgrade, Serbia

**Keywords:** Tubular functional capacity, effective renal plasma flow, glomerular filtration rate, intact parathyroid hormone, chronic kidney failure

## Abstract

Clinical data regarding the interaction between tubular functional capacity (TFC) and maladaptive parathyroid gland response in early-stage chronic kidney disease (CKD) are limited. This study aimed to evaluate the association between parathyroid gland response, measured as intact parathyroid hormone (iPTH) serum concentration (pg/mL) using chemiluminescent microparticle immunoassay, and the dissociation between the decline in glomerular filtration rate (GFR) and TFC, assessed through radionuclide clearances. TFC was evaluated by measuring effective renal plasma flow (mERPF, mL/min/1.73m^2^) using (131I) Hippurate (131I-H) clearance, while GFR was measured using (99m) Tc-DTPA (mGFR, mL/min/1.73m^2^). Consecutive participants with preexisting CKD (*N* ═ 111, female 44%, male 56%) were enrolled and stratified into four groups based on CKD stages (1, 2, 3a, and 3b). Median serum iPTH concentrations significantly differed between Stage 1 [23 (20.4–25.5) pg/mL] and Stage 2 [23.6 (20.5–26.8) pg/mL] compared to Stage 3a [38.1 (34.1–41.9) pg/mL] and Stage 3b [45.8 (39.7–51.9) pg/mL] (*P* ═ 0.01). In Stage 1, there was a significant positive association between iPTH and mERPF (*P* ═ 0.003). Conversely, in Stage 3b, iPTH was significantly negatively associated with both mGFR and mERPF (*P* < 0.05 for both). Regression models that included the interaction between CKD stage and either mGFR or mERPF, alongside other predictors (age, CKD stage, body mass index, ionized calcium, and 25-hydroxyvitamin D), revealed significant associations with iPTH (*P* < 0.05 for all variables). The assessment of TFC using 131I-H plasma clearance does not enhance the detection of maladaptive parathyroid gland responses compared to evaluating CKD stage and its relationship with declining glomerular and tubular clearances in early-stage CKD patients.

## Introduction

At present, hyperparathyroidism (HPT) is a relatively common condition, frequently identified through biochemical screening in the absence of overt clinical signs of parathyroid gland disease. The clinical profiles of HPT encompass primary (including asymptomatic, normocalcemic variants and hypercalcemic HPT), regulatory, secondary (SHPT), and tertiary (THPT) forms, all characterized by excessive secretion from the parathyroid glands and downregulation of the calcium-sensing receptor (CaSR). These characteristics arise from various etiological factors, including genetic and epigenetic changes [1, 2]. While primary and tertiary HPT have clear etiologies [3–5], regulatory and secondary HPT are typically consequences of prolonged hyperphosphatemia and hypocalcemia, resulting from multiple metabolic and target organ dysfunctions, including renal, bone, gastrointestinal, neurological, and hepatic diseases [6].

The complexity of evaluating the causes and effects of chronic kidney disease (CKD), particularly in the elderly, presents opportunities for a deeper understanding of the mechanisms underlying increased maladaptive parathyroid hormone (PTH) secretion. This understanding broadens the perspective on the impact of SHPT on bone metabolic abnormalities, heterotopic vascular and soft tissue calcifications, cardiovascular events, and mortality [7]. Regardless of the underlying cause of CKD, a decline in glomerular filtration rate (GFR) has numerous consequences and is regarded as a pivotal factor driving the maladaptive response of the parathyroid glands in SHPT. Early-stage CKD, defined as stages 1–3, is often asymptomatic and frequently undiagnosed [8]. Disruption in PTH secretion begins in the early stages of CKD, influenced by the interplay between calcium, phosphate, active vitamin D, and fibroblast growth factor 23 (FGF23) [9]. After an initial decrease in FGF23 and impaired feedback mechanisms in the parathyroid glands, serum PTH concentrations start to rise when the GFR falls below 60 mL/min/1.73m^2^ [10]. Furthermore, the kidneys and liver play crucial roles in the impaired renal clearance of PTH, contributing to SHPT in CKD [11]. Research on renal extraction of PTH has highlighted the importance of peritubular clearance, a process that clears the majority of PTH (1–84) and its fragments from circulation, independent of GFR [12, 13]. Given the theoretical possibility that renal tubular injury could precede glomerular injury—and that significant decrements in tubular function can occur even with “normal” GFR—the characterization of tubular functional capacity (TFC) may serve as a valuable tool for evaluating PTH levels in the early stages of CKD [14, 15].

The present study, with its rigorous design, aimed to investigate the association between parathyroid gland response and the dissociation between declines in both GFR and TFC, assessed via radionuclide clearances. Estimating TFC using effective renal plasma flow (ERPF), a crucial component of renal blood flow that supplies the kidney’s secretory structures, enhances the reliability and validity of the findings.

## Materials and methods

### Study subjects and protocol

An observational, cross-sectional study was conducted at the University Clinical Center of Vojvodina (UCCV). Consecutive participants were enrolled and referred to the Department of Laboratory Diagnostics and Nuclear Medicine at UCCV for kidney function assessment using radionuclide renal clearances. Patients with previously diagnosed CKD due to chronic tubulointerstitial diseases were evaluated for baseline kidney function prior to the initiation of potentially nephrotoxic therapies. A standardized protocol for all patients undergoing radionuclide renal clearances included two visits. During the first visit, radionuclide renal clearance for measuring GFR (mGFR) and venous blood sampling for laboratory analysis were performed between 7 AM and 9 AM while fasting for 12 h after the last meal. The second visit, occurring within seven days of the first (provided there was no change in the patient’s clinical status), involved radionuclide renal clearance for measuring ERPF (mERPF). Anthropometric measurements—including body height (BH), body weight (BW), and waist circumference (WC)—were taken at both visits, and body mass index (BMI = BW/BH^2^, kg/m^2^) and body surface area (BSA) were calculated. Patients provided two urine samples: a 24-h collection during the first visit and a first-morning sample during the second visit. If both results fell within the same albuminuria category, the patient data were included in subsequent analyses.

The study included both genders, with female participants (49/111, 44%) and male participants (62/111, 56%). Patients were classified into GFR categories G1, G2, G3a, and G3b, with kidney damage indicated by albuminuria in categories A1 and A2 [16]. Patients were stratified into four groups: Stage 1 (mGFR ≥90 mL/min/1.73m^2^ with kidney damage, *N* ═ 25), Stage 2 (mGFR 60–89 mL/min/1.73m^2^ with kidney damage, *N* ═ 30), Stage 3a (mGFR 45–59 mL/min/1.73m^2^, *N* ═ 26), and Stage 3b (mGFR 30–44 mL/min/1.73m^2^, *N* ═ 30). Patients with albuminuria category A3 (AER>300 mg/24 h) were excluded from further testing due to methodological constraints and the risk of overestimating expanded body space in cases of severe albuminuria. The study also excluded patients with CKD stages 4 and 5, diabetes mellitus, liver and gastrointestinal diseases, inflammatory, autoimmune, and infectious diseases, endocrine gland dysfunctions, malignancies, individuals exhibiting clinically evident edema or ascites, pregnancy, and those on routine medications that could affect PTH levels (including corticosteroids, estrogen replacement, biotin supplementation, diuretics, sodium-glucose cotransporter-2 inhibitors, lithium, anticonvulsants such as phenytoin/phenobarbital, bisphosphonates, denosumab, romosozumab, calcitonin, and calcium channel blockers).

### Radionuclide renal clearance

Radionuclide renal clearance methods were conducted in accordance with established standard operating procedures [17, 18] and local protocols. Following hydration with water (5 mL/kg body mass), plasma clearances were obtained using a single injection of a commercially available radiopharmaceutical. Blood sampling for GFR was scheduled for 180 and 240 min after the intravenous injection of 30 MBq technetium-99m pentetate (DTPA) (Technescan DTPA, Mallinckrodt Medical B.V., Netherlands). ERPF was assessed 20 and 30 min after intravenous administration of 1 MBq Hippurate (131I-H) (Institute for Nuclear Science, Vinca, Serbia). Venous blood samples were collected from the contralateral limb to minimize contamination risk. The radioactivity of plasma samples was quantified using a gamma counter equipped with a NaI(Tl) crystal (Captus 3000 by Capintec, USA), with appropriate energy windows for technetium-99m and iodine-131. Daily quality control was performed per the manufacturer’s recommendations. Calculations for mGFR and mERPF were performed using the slope-intercept method [Captus 3000 Built-in Software, version 1.28 (2013)]. The Brochner-Mortensen correction was applied for mGFR calculations, while mERPF was adjusted by multiplying by 0.8 to account for a 20% overestimation [19]. Obtained values were normalized to BSA using the DuBois and DuBois formula [20]. Corrected mGFR and mERPF values were then compared to age- and sex-specific reference values (ASS GFR and ASS ERPF) [21], and absolute and relative deviations for age- and sex-specific GFR (DEV ASS GFR, mL/min/1.73m^2^, %) as well as absolute and relative deviations for age- and sex-specific ERPF (DEV ASS ERPF, mL/min/1.73m^2^, %) were calculated.

ASS GFR: 144.1 -- (0.99 × age) for females; ASS GFR: 160.5 -- (1.16 × age) for males

ASS ERPF: 673.3 – (2.92 × age) for females; ASS ERPF: 854.2 -- (5.4 × age) for males

Chromatography was performed to ensure the quality control of radiochemical purity (RCP): paper chromatography for technetium-99m DTPA with RCP >95%; thin-layer chromatography for Hippurate (131I-H) with RCP ≥96%; and radiochemical impurities: % (131I) ≤2% and 2-iodo (131I) benzoic acid ≤2%.

### Biochemical analyses

Serum concentrations of glucose, urea, creatinine, uric acid, total calcium (tCa), ionized calcium (iCa), phosphorus (P), and magnesium (Mg) were measured using spectrophotometric methods on an automated analyzer (Alinity c, Abbott). Serum iCa was determined using an ion-selective electrode method on the AVL 9180 analyzer (Roche Diagnostics). Lipid profile parameters, including total cholesterol (tChol), triglycerides (TG), and high-density lipoprotein cholesterol (HDL-C), were assessed spectrophotometrically using the Alinity c analyzer. Low-density lipoprotein cholesterol (LDL-C) was calculated using Friedewald’s formula. Apolipoproteins Apo AI and Apo B were quantified via immunoturbidimetric assay. Serum cystatin C (CysC) concentrations were measured using the nephelometric method on the BN ProSpec System (Siemens). The albuminuria excretion rate (AER, mg/24 h) was determined in 24-h urine samples using an immunoturbidimetric method on the Alinity c analyzer. In spot urine samples, albumin and creatinine concentrations were measured, and the albumin-to-creatinine ratio (ACR, mg/mmol) was calculated.

Serum concentrations of insulin, 25-hydroxyvitamin D [25(OH)D], and intact PTH (iPTH) were analyzed using chemiluminescent microparticle immunoassay (CMIA) on the Alinity i analyzer (Abbott). Vitamin D deficiency was defined as serum 25(OH)D levels below 50 nmol/L [22]. The insulin resistance index (Homeostasis Model Assessment – Insulin Resistance, HOMA-IR) was calculated for all participants [23]. The reference range for iPTH in adults was 15–68.3 pg/mL. Internal laboratory quality control was conducted to assess the measurement of iPTH serum concentration. iPTH levels were monitored over 20 days using control levels provided by three manufacturers (low, medium, and high control; iPTH STAT Protocol, Abbott) and two different iPTH concentrations in blood samples (pool serum). Imprecision (CV total) and bias (absolute and relative) were consistent with the manufacturer’s specifications: low control 8.3 pg/mL (CV 4.4%, Bias 0.67; Bias % 8.08%); medium control 54.2 pg/mL (CV 3.9%; Bias 1.85; Bias % 3.41%); high control 208.3 pg/mL (CV 4.0%, Bias 7.75; Bias % 3.72%); I pool - 74.8 pg/mL (CV 3.2%, Bias --8.40; Bias % 11.23%); II pool 156.2 pg/mL (CV 4.2%, Bias --22.56; Bias % 14.44%).

### Ethical statement

The study was conducted in accordance with the guidelines of the Declaration of Helsinki and was approved by the Ethics Committee of UCCV (No. 00-347/2025). All participants provided written informed consent prior to their involvement in the study. All methods were executed in compliance with relevant guidelines and regulations.

### Statistical analysis

The distribution of variables was evaluated using the Shapiro–Wilk test. Continuous variables with a normal distribution are presented as mean ± standard deviation, while non-normally distributed variables are reported as median with lower and upper quartiles (Q1–Q3). Depending on data distribution, ANOVA or the Kruskal–Wallis test was employed for multiple-group comparisons, followed by a Bonferroni post hoc test. Spearman’s rank correlation coefficient was calculated to describe monotonic relationships between iPTH serum concentrations and other continuous variables. Scatter plots were utilized to visualize relationships between iPTH and independent variables (renal clearance measurements: mGFR and mERPF) within the study group. Univariate linear regression analyses were initially performed for each stage 1–3b group to explore the relationships between iPTH and renal clearances. Subsequently, multivariate linear regression models with interactions were employed to examine the relationship between iPTH and variables associated with kidney function. The variables assessed included renal clearance measurements, CKD stage, and interactions between CKD stage and mGFR, and mERPF. To mitigate confounding between mGFR and mERPF, each variable was analyzed in separate models (GFR model and ERPF model). Finally, multivariate linear regression models were used to identify predictors of iPTH serum concentration. CysC and phosphorus serum concentrations were excluded due to their lack of significant effects in initial model analyses. Model 1 included CKD stage, interaction (CKD stage × mGFR), age, BMI, iCa, and 25(OH)D; Model 2 included CKD stage, interaction (CKD stage × mERPF), age, BMI, iCa, and 25(OH)D. Residuals were analyzed for both models, and collinearity was assessed using tolerance. Models were compared using Akaike’s information criterion (AIC). Statistical significance was set at an alpha level of 0.05. Analyses were conducted using statistical software Stata 18 (StataCorp LLC, 2023) and Statistica 14.0.0.15 (TIBCO Software Inc.).

## Results

Table 1 presents a comprehensive comparison of clinical, metabolic, and mineral profiles across various patient groups. Patients in CKD Stages 3a and 3b exhibited significantly higher median ages compared to those in Stages 1 and 2 (*P* < 0.001). No statistically significant differences were observed in anthropometric measurements (BMI and WC) or blood pressure values (SP, DP, MAP) among the groups. Analysis of laboratory parameters revealed significant differences in median levels of glucose and lipids, including HDL-C, TG, and apo AI (all *P* < 0.05). Conversely, no significant differences were detected in the median serum concentrations of electrolytes (iCa, P, Mg) or 25(OH)D. Among all patients, 47% exhibited concurrent vitamin D deficiency. The median serum concentrations of iPTH in the Stage 1 and Stage 2 groups (23 [20.4–25.5] vs 23.6 [20.5–26.8] pg/mL, *P* > 0.05) were significantly lower (*P* ═ 0.01) than those in the Stage 3a and Stage 3b groups (38.1 [34.1–41.9] vs 45.8 [39.7–51.9] pg/mL, *P* > 0.05). Renal function profile parameters demonstrated consistent statistical significance across groups (Table 2). Median CysC serum concentrations did not differ significantly between Stages 1 and 2 but were significantly lower in Stages 1 and 2 compared to Stages 3a and 3b. Both mGFR and mERPF values declined significantly with each advancing stage (*P* < 0.05 for both). DEV ASS GFR (%) and DEV ASS ERPF (%) exhibited statistically significant differences across all stages (*P* < 0.05 for both).

**Table 1:** Clinical, metabolic, and mineral profiles of patients

**Variables**	**Stage 1**	**Stage 2**	**Stage 3a**	**Stage 3b**	***P***
	***n* ═ 25**	***n* ═ 30**	***n* ═ 26**	***n* ═ 30**	
Age (years)	41^a^ (18–66)	48^a^ (22–70)	65^b^ (43–82)	65^b^ (22–77)	0.00
Male, *N*/total	16/25	16/30	10/26	20/30	0.14
BMI (kg/m^2^)	27.4 ± 3.5	26.8 ± 5.1	27.6 ± 3.8	28.9 ± 4.2	0.29
WC (cm)	97.5 ± 11.9	91.2 ± 14.7	95.7 ± 11.2	99.1 ± 12.2	0.12
SP (mm Hg)	125.8 ± 13.4	130.2 ± 22.9	118.5 ± 29.3	139.6 ± 19.7	0.66
DP (mm Hg)	79.4 ± 7.9	80.8 ± 12.1	80.4 ± 7.4	81.1 ± 9.8	0.87
MAP (mm Hg)	94.8 ± 8.8	97.3 ± 15.5	94.9 ± 12.6	97.3 ± 12.3	0.72
Glucose (mmol/L)	5.3^a^ (4.6–5.5)	5.01^a^ (4.7–5.4)	5.4^a,b^ (4.98–6.1)	5.6^b^ (5.3–6.1)	0.01
Insulin (mIU/L)	19 (9.7–25)	11.8 (8.2–15.5)	11.7 (7.8–17.8)	14.4 (11–32.3)	0.12
HOMA-IR	4.5 (2.2–6.2)	2.85 (1.8–3.7)	2.65 (1.7–4.9)	3.2 (2.4–7.8)	0.09
tChol (mmol/L)	5.4 (3.9–5.8)	5.2 (4.5–6.3)	5.4 (4.4–5.8)	4.7 (4.2–5.7)	0.18
LDL-C (mmol/L)	3.7 (2.3–3.8)	3.2 (2.4–4.1)	3.3 (2.8–3.6)	2.8 (2.3–3.3)	0.12
HDL-C (mmol/L)	1.3^a^ (1.1–1.6)	1.3^a^ (1–1.6)	1.3^a^ (1.2–1.5)	1.2^b^ (0.9–1.4)	0.04
TG (mmol/L)	1.1^a^ (0.9–1.4)	1.6^a,b^ (1.1–2.2)	1.5^a,b^ (1–1.9)	1.6^b^ (1.1–2)	0.01
Apo AI (mmol/L)	1.4^a^ (1.3–1.6)	1.5^a^ (1.3–1.7)	1.5^a^ (1.4–1.7)	1.3^b^ (1.0–1.4)	0.01
Apo B (mmol/L)	1.1 (0.9–1.2)	1 (0.8–1.3)	1.1 (0.9–1.2)	1 (0.9–1.2)	0.66
tCa (mmol/L)	2.4 (2.3–2.5)	2.5 (2.4–2.5)	2.4 (2.3–2.5)	2.4 (2.3–2.5)	0.25
iCa (mmol/L)	1.1 (1.07–1.1)	1.1 (1.1–1.2)	1.1 (1.1–1.2)	1.1 (1–1.3)	0.59
P (mmol/L)	1.03 (0.9–1.1)	1.1 (0.9–1.1)	1.1 (0.9–1.1)	1.1 (0.8–1.1)	0.74
Mg (mmol/L)	0.8 (0.8–0.9)	0.8 (0.8–0.9)	0.8 (0.7–0.9)	0.8 (0.7–0.9)	0.16
iPTH (pg/mL)	23^a^ (20.4–25.5)	23.6^a^ (20.5–26.8)	38.1^b^ (34.1–41.9)	45.8^b^ (39.7–51.9)	0.01
25(OH)D (nmol/L)	51.4 (42.5–60.3)	44.6 (37.9–51.2)	49.7 (40.3–59.1)	53.3 (47.3–59.1)	0.36

Groups designated by the same letter (a, b, c) do not exhibit significant differences in the post-hoc testing. Abbreviations: BMI: Body mass index; WC: Waist circumference; SP: Systolic pressure; DP: Diastolic pressure; MAP: Mean arterial pressure; HOMA-IR: Homeostasis model assessment of insulin resistance; tChol: Total cholesterol; LDL-C: Low-density lipoprotein cholesterol; HDL-C: High-density lipoprotein cholesterol; TG: Triglycerides; Apo A-1: Apolipoprotein A-1; Apo B: Apolipoprotein B; tCa: Total serum calcium concentration; iCa: Ionized serum calcium concentration; P: Serum phosphorus concentration; Mg: Serum magnesium concentration; iPTH: Intact parathyroid hormone serum concentration; 25(OH)D: Serum 25-hydroxyvitamin D concentration.

**Table 2:** Renal function profile of patients

**Variables**	**Stage 1**	**Stage 2**	**Stage 3a**	**Stage 3b**	***P***
	***n* ═ 25**	***n* ═ 30**	***n* ═ 26**	***n* ═ 30**	
CysC (mg/L)	0.8^a^ (0.7–1)	1^a^ (0.9–1.1)	1.3^b^ (1.1–1.5)	1.8^c^ (1.4–1.9)	0.01
Creatinine (µmol/L)	88^a^ (55.7–90.5)	81^a,b^ (70–104)	100^b^ (87–118)	125^b,c^ (109–155)	0.01
Urea (mmol/L)	5.2^a^ (4.5–5.4)	5.1^a,b^ (4.6–6.7)	6.7^b^ (5.3–8.1)	8.8^c^ (7.6–10.2)	0.01
Uric acid (µmol/L)	347^a^ (279–396)	337^a^ (283–386)	370^a,b^ (301–406)	423^b^ (371–462)	0.01
ACR (mg/mmol)	0.7 (0.7–1.8)	1.75 (1.33–6.2)	1.1 (0.8–2.9)	3.2 (1–8.6)	0.12
AER (mg/24h)	9.3 (7.8–20)	13.9 (6–89)	6.9 (3.2–31)	16 (5–81)	0.18
mGFR (mL/min/1.73m^2^)	96^a^ (94–98.2)	77^b^ (72–84)	52^c^ (48–56)	38.5^d^ (35–41.9)	0.01
ASS-GFR (mL/min/1.73m^2^)	112.9^a^ (110.3–120.2)	104.8^a^ (90.9–119.9)	84.5^b^ (79.3–99)	85.1^b^ (80.5–95.5)	0.01
DEV ASS GFR (mL/min/1.73m^2^)	17.9^a^ (14.8–22.9)	29.3^b^ (21–41.1)	32^b^ (29.1–42.2)	49.6^c^ (43–55.6)	0.01
DEV ASS GFR (%)	16^a^ (11–21)	26.7^b^ (22.4–33.2)	40^c^ (37–43)	56.7^d^ (52–60)	0.01
mERPF (mL/min/1.73m^2^)	520^a^ (510–534)	436^b^ (351–490)	298.5^c^ (255–313)	235^d^ (217–260)	0.01
ASS-ERPF (mL/min/1.73m^2^)	633^a^ (627–665)	595^a^ (530–665)	500^b^ (476–568)	503^b^ (482–552)	0.01
DEV ASS ERPF (mL/min/1.73m^2^)	135^a^ (30–146)	166^b^ (129–193)	221^b^ (173–276)	279^c^ (225–325)	0.01
DEV ASS ERPF (%)	20^a^ (10–23)	26^b^ (22–31)	46^c^ (36–51)	55^d^ (46–59)	0.01

Groups denoted by the same letter (a, b, c, d) do not exhibit significant differences in the post-hoc testing. Abbreviations: Cys C: Cystatin C; AER: Albumin excretion rate; ACR: Albumin-to-creatinine ratio; mGFR: Measured glomerular filtration rate; ASS-GFR: Age- and sex-specific glomerular filtration rate; DEV ASS GFR: Deviation for age- and sex-specific glomerular filtration rate; mERPF: Measured effective renal plasma flow; ASS-ERPF: Age- and sex-specific effective renal plasma flow; DEV ASS ERPF: Deviation for age- and sex-specific effective renal plasma flow.

**Table 3:** Correlation between iPTH and variables (*n* ═ 111)

**Variable**	**r**	***P***
Age (years)	0.42	<0.01
WC (cm)	0.22	0.02
BMI (kg/m^2^)	0.27	0.01
MAP (mm/Hg)	0.14	0.15
Apo B (mmol/L)	0.10	0.26
LDL-C (mmol/L)	--0.05	0.59
HOMA IR	0.15	0.10
iCa (mmol/L)	--0.04	0.65
P (mmol/L)	--0.04	0.41
25 (OH)D (nmol/L)	--0.19	0.04
Cys C (mg/L)	0.48	<0.01
mGFR (mL/min/1.73m^2^)	--0.66	<0.01
ASS-GFR (mL/min/1.73m^2^)	--0.43	<0.01
DEV-ASS GFR (%)	0.51	<0.01
mERPF (mL/min/1.73m^2^)	--0.68	<0.01
ASS-ERPF (mL/min/1.73m^2^)	--0.43	<0.01
DEV-ASS ERPF (%)	0.57	<0.01

Abbreviations: WC: Waist circumference; BMI: Body mass index; MAP: Mean arterial pressure; HOMA-IR: Homeostasis model assessment of insulin resistance; LDL-C: Low-density lipoprotein cholesterol; Apo B: Apolipoprotein B; iCa: Ionized serum calcium concentration; 25(OH)D: Serum 25-hydroxyvitamin D concentration; mGFR: Measured glomerular filtration rate; ASS-GFR: Age- and sex-estimated glomerular filtration rate; DEV ASS GFR: Deviation for age- and sex-estimated glomerular filtration rate; mERPF: Measured effective renal plasma flow; ASS-ERPF: Age- and sex-specific effective renal plasma flow; DEV ASS ERPF: Deviation for age- and sex-estimated effective renal plasma flow; r: Spearman correlation coefficient; P: Level of significance.

**Figure 1.**
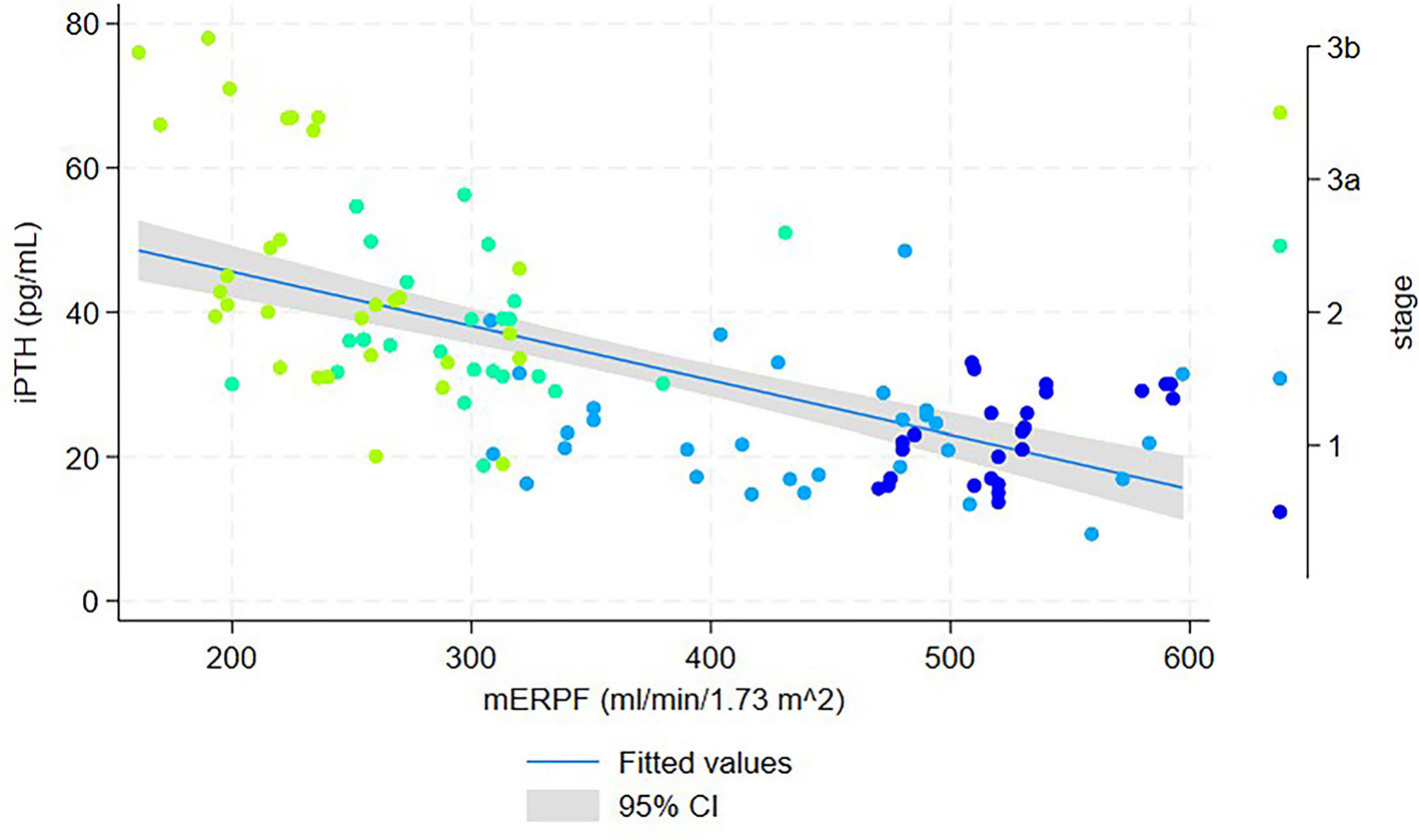
**Scatter plot of serum iPTH vs mERPF in patients with early-stage CKD (*n* ═ 111).** Each point represents an individual participant and is color-coded by chronic kidney disease stage (1-3b). The solid line shows the fitted values from a linear regression model (iPTH = 60.67 – 0.08 × mERPF), and the shaded area denotes the 95% confidence interval. Abbreviations: iPTH: Intact parathyroid hormone; mERPF: Measured effective renal plasma flow; CKD: Chronic kidney disease; CI: Confidence interval.

Spearman correlation analysis (Table 3) revealed statistically significant positive monotonic relationships between iPTH and age (*r* ═ 0.42, *P* < 0.01), WC (*r* ═ 0.22, *P* ═ 0.02), BMI (*r* ═ 0.27, *P* ═ 0.01), and CysC (*r* ═ 0.48, *P* < 0.01). Statistically significant negative monotonic relationships were observed between iPTH and 25(OH)D (r ═ –0.19, *P* ═ 0.04), mGFR (r ═ –0.66, *P* < 0.01), and mERPF (*r* ═ –0.68, *P* < 0.01).

**Figure 2.**
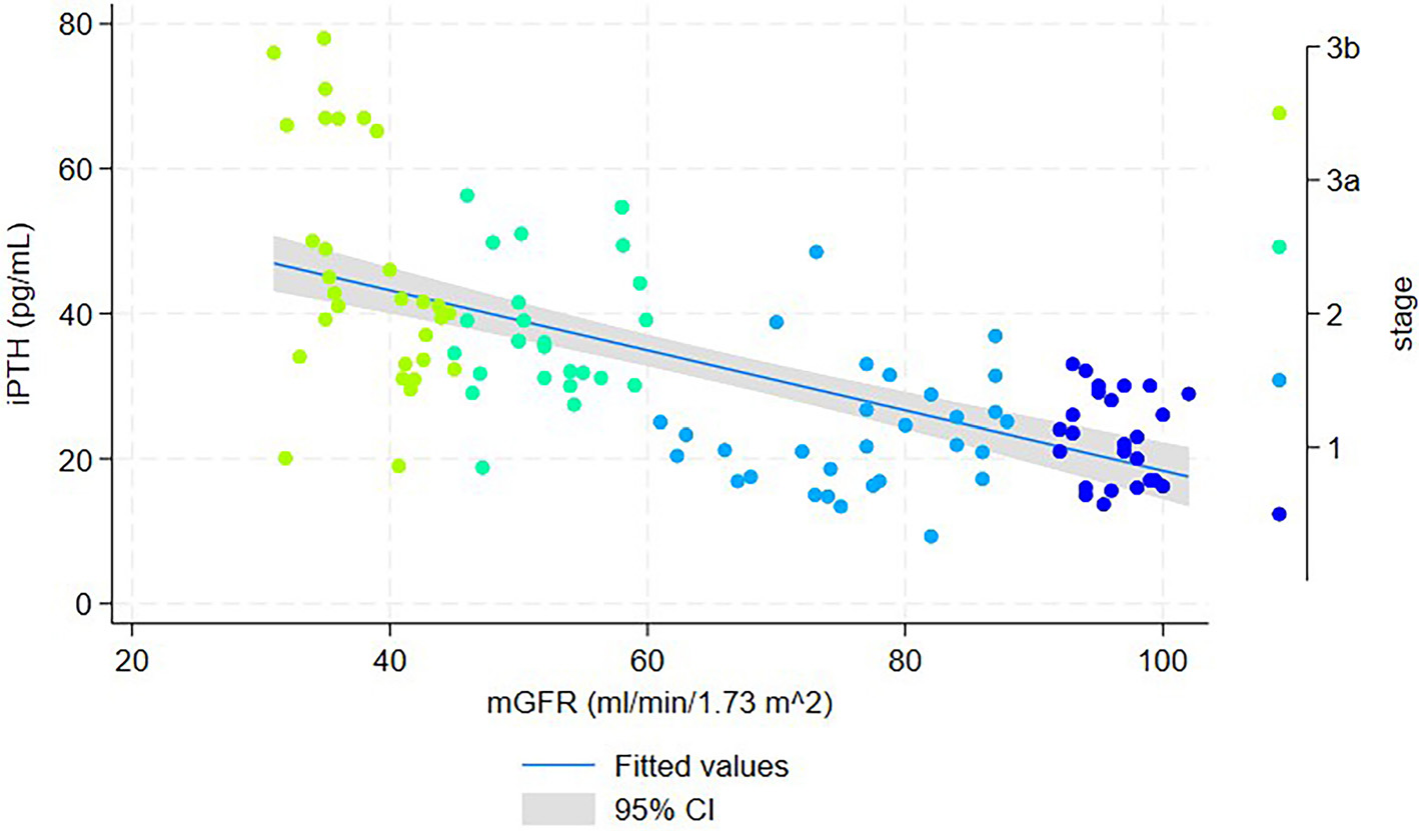
**Scatter plot of serum iPTH vs mGFR in patients with early-stage CKD (*n* ═ 111).** Each point represents an individual participant and is color-coded by CKD stage (1-3b). The solid line shows fitted values from a linear regression model (iPTH = 59.75 – 0.41 × mGFR), and the shaded area denotes the 95% CI. Abbreviations: iPTH: Intact parathyroid hormone; mGFR: Measured glomerular filtration rate; CKD: Chronic kidney disease; CI: Confidence interval.

In the cohort of 111 patients, a scatter plot with fitted regression lines and 95% confidence intervals illustrated a linear relationship between iPTH and mERPF (Figure 1), with the regression equation iPTH = 60.67–0.08 * mERPF. Figure 2 further demonstrates a linear relationship between iPTH and mGFR, with the regression equation iPTH = 59.75–0.41 * mGFR.

Table 4 summarizes the univariate linear regression results examining the relationship between serum iPTH concentration and renal clearance measurements (mGFR and mERPF) across Stages 1 to 3b. Group analyses revealed a significant negative linear association between mGFR and iPTH in Stage 3b (*P* ═ 0.007). Additionally, in CKD Stage 3b, a significant negative linear association between mERPF and iPTH was observed (*P* ═ 0.001). In Stage 1, mERPF and iPTH showed a significant positive association (*P* ═ 0.003).

Table 5 summarizes the relationship between serum iPTH concentration and variables associated with kidney function. In the GFR model (*R*^2^ ═ 0.68, adjusted *R*^2^ ═ 0.46, *P* < 0.001), both CKD stage (β ═ 0.922, *P* ═ 0.01) and the interaction term (CKD stage x mGFR) (β ═ –0.297, *P* ═ 0.006) were significantly associated with serum iPTH concentration. In the ERPF model (*R*^2^ ═ 0.72, adjusted *R*^2^ ═ 0.52, *P* < 0.001), CKD stage (β ═ 1.423, *P* < 0.001) and the interaction term (CKD stage × mERPF) (β ═ –0.605, *P* < 0.001) were also significantly associated with serum iPTH concentration.

The results of the multivariate regression models are presented in Table 6. In Model 1 (*R*^2^ ═ 0.79, adjusted *R*^2^ ═ 0.63, *P* < 0.001; AIC = 814.645), CKD stage (β ═ 0.978, *P* < 0.001), the interaction term (CKD stage x mGFR) (β ═ –0.347, *P* < 0.001), age (β ═ –0.157, *P* < 0.001), BMI (β ═ 0.202, *P* < 0.001), iCa (β ═ –0.228, *P* < 0.001), and 25(OH)D (β ═ –0.325, *P* < 0.001) were each independently and significantly associated with serum iPTH concentration. In this model, each increase in CKD stage was associated with a 12.85 pg/mL increase in serum iPTH concentration.

**Table 4:** Univariate linear regression analysis of iPTH serum concentration and renal clearance measurements (mGFR and mERPF) among groups

**iPTH (pg/mL)**					
**Stage 1 (mGFR ≥90 mL/min/1.73m^2^ with kidney damage, *n* = 25)**					
	**Unstandardized coefficients**		**95% CI**		
mGFR (mL/min/1.73m^2^)	B	SE(β)	Lower bound	Upper bound	*P*
Slope	--0.25	0.45	--1.18	0.69	0.594
Intercept	46.59	43.77	--43.96	137.14	0.298
mERPF (mL/min/1.73m^2^)	B	SE(β)	Lower bound	Upper bound	*P*
Slope	0.09	0.03	0.03	0.15	0.003
Intercept	--26.41	15.12	--57.67	4.88	0.094
**Stage 2 (mGFR 60–89 mL/min/1.73m^2^ with kidney damage, *n* = 30)**					
	**Unstandardized coefficients**		**95% CI**		
mGFR (mL/min/1.73m^2^)	B	SE(β)	Lower bound	Upper bound	*P*
Slope	0.12	0.19	--0.28	0.52	0.549
Intercept	14.44	15.23	--16.76	45.64	0.351
mERPF (mL/min/1.73m^2^)	B	SE(β)	Lower bound	Upper bound	
Slope	--0.01	0.02	--0.05	0.02	0.469
Intercept	29.61	8.31	12.59	46.63	0.001
**Stage 3a (mGFR 45–59 mL/min/1.73m^2^, *n* = 26)**					
	**Unstandardized coefficients**		**95% CI**		
mGFR (mL/min/1.73m^2^)	B	SE(β)	Lower bound	Upper bound	*P*
Slope	0.32	0.41	--0.53	1.18	0.441
Intercept	21.15	21.68	--23.59	65.89	0.34
mERPF (mL/min/1.73m^2^)	B	SE(β)	Lower bound	Upper bound	*P*
Slope	--0.01	0.04	--0.92	0.08	0.918
Intercept	39.38	12.57	13.43	65.32	0.005
**Stage 3b (mGFR 30–44 mL/min/1.73m^2^, *n* = 30)**					
	**Unstandardized coefficients**		**95% CI**		
mGFR (mL/min/1.73m^2^)	B	SE(β)	Lower bound	Upper bound	*P*
Slope	--1.85	0.64	--3.16	--0.55	0.007
Intercept	116.88	24.58	66.52	167.24	<0.001
mERPF (mL/min/1.73m^2^)	B	SE(β)	Lower bound	Upper bound	*P*
Slope	--0.22	0.06	--0.33	--0.11	0.001
Intercept	97.95	13.62	70.1	125.84	<0.001

Abbreviations: IPTH: Intact parathyroid hormone serum concentration (pg/mL); mGFR: Measured glomerular filtration rate (mL/min/1.73m^2^); mERPF: Measured effective renal plasma flow (mL/min/1.73m^2^); B: Unstandardized coefficient: S.E._B: Standard error of B; P: Level of significance; 95% CI: 95% confidence interval for the unstandardized coefficient.

**Table 5:** Linear regression models analyzing the relationship between iPTH and variables related to renal clearance

	**iPTH (pg/mL)**					
**Variable**	**Standardized coefficients**		**Unstandardized coefficients**			
GFR-model	β	SE(β)	B	SE(β)	*P*	T
Intercept			20.280	21.839	0.355	
CKD stage	0.922	0.354	12.115	4.645	0.01	0.04
Interaction (CKD stage x mGFR)	--0.297	0.106	--0.156	0.056	0.006	0.449
mGFR (mL/min/1.73m^2^)	0.088	0.319	0.057	0.206	0.782	0.050
ERPF-model	β	SE(β)	B	SE(β)	*P*	T
Intercept			--0.481	13.865	0.972	
CKD stage	1.423	0.298	18.699	3.909	<0.001	0.051
Interaction (CKD stage x mERPF)	--0.605	0.146	--0.041	0.010	<0.001	0.212
mERPF (ml/min/1.73m^2^)	0.441	0.236	0.052	0.028	0.065	0.081

Abbreviations: iPTH: Intact parathyroid hormone serum concentration (pg/mL); CKD stage: Chronic kidney disease stage; mGFR: Measured glomerular filtration rate (mL/min/1.73m^2^); mERPF: Measured effective renal plasma flow (mL/min/1.73m^2^); β: Standardized coefficient; S.E._β: Standard error of β; B: Unstandardized coefficient; S.E._B: Standard error of B; *P*: Level of significance; T: Tolerance.

**Table 6:** Multiple linear regression models analyzing the relationship between iPTH and predictor variables

**Model 1** **AIC = 814.645**	**iPTH (pg/mL)**						
	**Standardized coefficients**		**Unstandardized coefficients**				
**Predictor variable**	**β**	**SE(β)**	**B**	**SE(β)**	**t-score**	***P***	**T**
Intercept			76.374	16.264	4.696	<0.001	
CKD stage	0.978	0.092	12.847	1.213	10.591	<0.001	0.419
Interaction (CKD stage x mGFR)	--0.347	0.083	--0.182	0.043	--4.184	<0.001	0.520
Age (years)	--0.157	0.074	--0.145	0.068	--2.127	<0.001	0.659
BMI (kg/m^2^)	0.202	0.063	0.698	0.217	3.218	<0.001	0.907
iCa (mmol/L)	--0.228	0.062	--44.917	12.142	--3.699	<0.001	0.945
25(OH)D (nmol/L)	--0.325	0.062	--0.244	0.046	--5.259	<0.001	0.934
							
**Model 2** **N = 111** **AIC = 807.216**	**iPTH (pg/mL)**						
	**Standardized coefficients**		**Unstandardized coefficients**				
**Predictor variable**	**β**	**SE(β)**	**B**	**SE(β)**	**t-score**	***P***	**T**
Intercept			56.777	14.112	4.023	<0.001	
CKD stage	1.007	0.088	13.232	1.154	11.466	<0.001	0.433
Interaction (CKD stage x mERPF)	--0.398	0.078	--0.027	0.005	--5.091	<0.001	0.546
Age (years)	--0.169	0.071	--0.156	0.066	--2.376	<0.001	0.660
BMI (kg/m^2^)	0.203	0.060	0.702	0.209	3.364	0.001	0.917
iCa (mmol/L)	--0.158	0.059	--31.242	11.697	--2.671	0.009	0.952
25(OH)D (nmol/L)	--0.323	0.059	--0.242	0.045	--5.432	<0.001	0.946

Abbreviations: iPTH: Intact parathyroid hormone serum concentration (pg/mL); AIC: Akaike’s information criterion; CKD stage: Chronic kidney disease stage; mGFR: Measured glomerular filtration rate (mL/min/1.73m^2^); mERPF: Measured effective renal plasma flow (mL/min/1.73m^2^); BMI: Body mass index; iCa: Ionized serum calcium concentration; 25(OH)D: Serum 25-hydroxyvitamin D concentration; β: Standardized coefficient; S.E._β: Standard error of β; B: Unstandardized coefficient; S.E._B: Standard error of B; *P*: Level of significance; T: Tolerance.

In Model 2 (*R*^2^ ═ 0.80, adjusted *R*^2^ ═ 0.65, *P* < 0.001; AIC = 807.216), CKD stage (β ═ 1.007, *P* < 0.001), the interaction term (CKD stage x mERPF) (β ═ –0.398, *P* < 0.001), age (β ═ –0.169, *P* < 0.001), BMI (β ═ 0.203, *P* ═ 0.001), iCa (β ═ –0.158, *P* ═ 0.009), and 25(OH)D (β ═ –0.323, *P* < 0.001) were independently and significantly associated with serum iPTH concentration. In Model 2, each increase in CKD stage was associated with a 13.23 pg/mL increase in serum iPTH concentration.

## Discussion

This study evaluates indicators of glomerular function and TFC through radionuclide clearances in patients with CKD, and their relationship to iPTH levels across early stages. The novelty of this research lies in the observation of TFC through the measurement of ERPF, which irrigates the secretory-active structures of the kidney [24, 25].

In summary, this study found a significant negative correlation between mGFR, mERPF, and iPTH in early-stage CKD. When analyzing CKD stages separately, a significant negative linear association was detected between iPTH and both mGFR and mERPF in Stage 3b (GFR 30–44 mL/min/1.73m^2^). In Stage 1 (mGFR ≥ 90 mL/min/1.73m^2^ with A1 or A2 categories), iPTH exhibited significant positive associations with mERPF. These findings underscore the role of tubular secretory pathways in regulating iPTH levels. In addition to measured clearances for glomerular and tubular function, this study found that TFC, measured by 131I-H plasma clearance, was not a significant predictor of iPTH levels in patients with early-stage CKD. Furthermore, a significant association was identified between serum iPTH concentration and the interaction between CKD stage and TFC. Similarly, within the model focusing on glomerular function, CKD stage and the interaction between CKD stage and GFR were independently and significantly associated with iPTH levels.

The prevalence of SHPT in CKD patients remains high, irrespective of diagnostic criteria. A recent study by Wang et al. [26] reported a prevalence of 49.5%. Most measured iPTH levels in our study fell within the reference range (15–68.3 pg/mL). Median iPTH levels did not significantly differ between Stage 1 and Stage 2; however, levels in Stages 1 and 2 were significantly lower than in Stages 3a and 3b. Additionally, our results indicated that each increase in CKD stage correlated with a 13 pg/mL increase in iPTH levels, regardless of whether the predictor was the interaction between CKD stage and GFR or TFC. These findings align with previous studies [7, 27], indicating a potential rise in iPTH levels as GFR declines below 60 mL/min/1.73m^2^. Current guidelines recommend monitoring iPTH levels in CKD Stage 3 patients when GFR falls below this threshold. Notably, the guidelines also indicate that the optimal PTH level for Stage 3 CKD patients remains undetermined, as does the reference range adjusted for age and 25(OH) vitamin D levels in this vulnerable population [16].

Furthermore, mERPF demonstrated statistically significant negative linear coefficients concerning iPTH levels in CKD stage 3b. There is a paucity of published studies exploring the association between ERPF as a potential indicator of TFC and iPTH levels. Clinical evaluation of tubular secretion as an early, independent marker is constrained by the absence of precise quantification methods, variability in patient hydration, concurrent pharmacotherapies, and the complexities associated with timed urine collections and fluctuating flow rates [25]. Physiologically, tubular secretion serves as the primary renal mechanism for eliminating most drugs and their metabolites, necessitating routine measurement. In humans, 131I-H clearance is approximately 15% lower than para-aminohippuric acid (PAH) clearance, the gold standard for mERPF [19, 28, 29].

According to Fine et al. [19], the correlation between ERPF derived from PAH clearance is notably high (*r* ═ 0.90, *P* < 0.01). However, PAH has not been widely adopted in clinical practice due to the complexity of its analytical procedures [30]. The pharmacokinetic characteristics of 131I-H closely resemble those of PAH. Following intravenous administration, the bolus rapidly enters renal circulation and reaches individual nephrons, where it is eliminated through both glomerular filtration (approximately 20%) and active tubular secretion (approximately 80%). Consequently, the excretion of 131I-H is directly proportional to the filtered plasma fraction. 131I-H enters Bowman’s space as primary urine, while the remaining plasma (approximately four-fifths) exits the glomeruli via efferent arterioles and enters the peritubular capillaries. Proximal tubule cells then actively uptake, transport, and secrete the residual 131I-H into the urine through energy-dependent mechanisms. As a result, nearly the entire quantity of 131I-H present in each blood volume is removed and excreted in urine during a single renal passage [29, 31]. A small fraction of renal blood flow, slightly exceeding 10%, supplies the medullary structures, hilus, and capsule of the kidney, which lack a transport system for the extraction of organic anions, including 131I-H. Smith et al. introduced the term ERPF to describe the portion of renal plasma flow that supplies the kidney’s secretory structures [28]. Extraction efficiency for 131I-H in the kidneys, according to various authors, ranges from 0.84–0.94 [29]. Additionally, 99mTc-ethylene dicysteine (99mTc-EC) is a significant agent for estimating renal tubular function. In both healthy individuals and patients, the plasma clearance of 99mTc-EC closely correlates with the clearance of 131I-H, averaging approximately 75% of 131I-H values. The renal extraction ratio for 99mTc-EC is 0.70 [32].

Diverse etiologies of CKD may influence the dissociation between the decline in GFR and TFC differently. Therefore, a widely available evaluation of GFR is insufficient for gaining deeper insights into early abnormalities. Tubular dysfunction often remains under-recognized and under-diagnosed in the earlier stages of CKD. In this study, ASS ERPF and ASS GFR values further refine result interpretation. Monitoring tubular function, in conjunction with GFR, in patients susceptible to SHPT, as well as elderly and vitamin D-deficient patients with CKD-related comorbidities (e.g., hypertension and diabetes), could delay the progression of SHPT and mitigate resistance to dietary, dialytic, or pharmacological therapies. These findings suggest that a comprehensive approach to CKD management, incorporating TFC monitoring, may be beneficial.

In our study, iCa levels emerged as a significant negative predictor of iPTH levels. Patients exhibited no abnormal changes in mineral profiles, including serum phosphate retention or hypocalcemia. These electrolyte imbalances are primary contributors to SHPT, characterized by a GFR below 60 mL/min per 1.73m^2^. Impairment in renal clearance of PTH during early CKD stages, attributable to decreased TFC (where renal peritubular uptake predominantly facilitates PTH 1–84 clearance from circulation), as evaluated through mERPF in this study, may represent one mechanism driving elevated iPTH levels [33]. Furthermore, the significant age-related functional declines in target organs (kidney, bone, intestine) coupled with dysregulated mineral homeostasis contribute to the development of SHPT [34]. Our findings corroborate previous research indicating that a decrease in mGFR and mERPF corresponds with a significant increase in serum iPTH concentrations in CKD stage 1–3b patients. Evidence suggests that maintaining phosphate and PTH levels within normal ranges is critical for preventing further deterioration of kidney function and cardiovascular incidence [9]. The multifactorial vulnerability of CKD patients with SHPT may elevate cardiovascular disease (CVD) mortality and morbidity by facilitating vascular calcification and promoting PTH 1 receptor (PTH1R) expression within the cardiovascular system [35]. Our study demonstrates that these patients exhibit elevated levels of proatherogenic lipid particles, reduced HDL-C, and increased TG levels as CKD stage advances.

We identified a statistically significant association between iPTH levels and biological determinants such as age, BMI, and 25(OH)D levels. Previous studies have highlighted biological factors influencing PTH levels; in addition to age, sex, and lifestyle factors (e.g., consumption of plant-based foods), genetic variants related to vitamin D metabolism, calcium, and renal phosphate transport account for 60% of variations in PTH levels [36, 37]. In our study, age and 25(OH)D levels were independently and significantly associated with iPTH levels in CKD patients, aligning with similar findings [37] and underscoring the relevance of these factors in interpreting results. Pulsatile fluctuations and circadian rhythms influence circulating PTH level variations, potentially impacting the accuracy of measured PTH concentrations. To address variability in iPTH levels resulting from intrinsic and extrinsic factors [38, 39], we analyzed laboratory parameters, implemented quality control measures, and assessed biological activity *in vivo*. Due to limited availability of EDTA plasma, iPTH was measured in serum samples, which may yield slightly lower iPTH concentrations than EDTA plasma due to peptide degradation during clotting, potentially introducing matrix-related bias.

The study is limited by a relatively small patient cohort, a restricted range of CKD etiologies focused on chronic tubulointerstitial diseases, and a reduced sample size due to time constraints. Additionally, incorporating measurements of other urinary biomarkers of renal tubular damage, such as kidney injury molecule-1 (KIM-1) and N-acetyl-ß-glucosaminidase (NAG) [40], may enhance the specificity of the observed association between TFC and iPTH levels. While radionuclide plasma clearance is utilized in this study to measure GFR and ERPF, all clearance methods are susceptible to systematic and random errors, which may lead to discrepancies between measured and actual renal function. Furthermore, biological conditions and analytical factors can fluctuate over time, contributing to variability in the measured parameters [40]. Notably, this study is the first to demonstrate a relationship between iPTH levels and mERPF in patients with early-stage CKD. Future investigations should concurrently assess both clearances, preferably employing clinically available, non-radioactive substances.

## Conclusion

The findings from this study suggest that evaluating TFC through 131I-H plasma clearance may aid in identifying declines in TFC in patients with early-stage CKD. However, assessing TFC via 131I-H plasma clearance does not enhance the detection of maladaptive parathyroid gland responses compared to evaluating CKD stage and its association with declining glomerular and tubular clearances in early-stage CKD patients.

## Data Availability

The data associated with the manuscript are available from the corresponding author upon reasonable request.
